# The interaction between MC4R gene variant (rs17782313) and dominant dietary patterns on depression in obese and overweight women: a cross sectional study

**DOI:** 10.1186/s12902-023-01335-0

**Published:** 2023-04-18

**Authors:** Mahya Mehri Hajmir, Atieh Mirzababaei, Cain C. T. Clark, Rasool Ghaffarian-Ensaf, Khadijeh Mirzaei

**Affiliations:** 1 Students’ Scientific Center, Tehran University of Medical Sciences (TUMS), PO Box 1417755331, Tehran, Iran; 2 Department of Community Nutrition, School of Nutritional Sciences and Dietetics, Tehran University of Medical Sciences (TUMS), P.O. Box:14155-6117, Tehran, Iran; 3 Centre for Intelligent Healthcare, Coventry University, Coventry, CV1 5FB UK; 4 Department of Nutrition, Science and Research Branch, Islamic Azad University, Tehran, Iran

**Keywords:** Obesity, Dominant food intake patterns, Depression, Melanocortin receptor 4 gene

## Abstract

**Background:**

Previous studies have shown that the minor allele (C allele) for melanocortin 4 receptor (MC4R) rs17782313 may be associated with depressed mood. Moreover, dietary patterns have potentially adverse effects on depression. This study investigates the interactions between the MC4R gene variant (rs17782313) and dietary patterns on depression among Iranian obese and overweight women.

**Methods:**

A total of 289 Iranian overweight and obese women, aged 18–50 years, were enrolled in this cross-sectional study. Biochemical, anthropometric, and body composition indices were assessed in all participants. Moreover, MC4R rs17782313, by the restriction fragment length polymorphism (PCR-RFLP) method, and depression, using the 21-item Depression Anxiety Stress Scales (DASS) questionnaire, were assessed. Food intakes were assessed by completing a 147-item semi-quantitative food frequency questionnaire (FFQ).

**Results:**

By the use of factor analysis, 2 major dietary patterns were extracted: healthy dietary pattern (HDP) and unhealthy dietary pattern (UDP). Binary logistic analysis showed that individuals with minor allele risk (CC) with high adherence to the unhealthy pattern increased odds for depression (OR: 8.77, 95%CI: -0.86-18.40, P: 0.07), after controlling for confounders. Also, a logical inverse relationship was observed between CT genotype and HDP on depression in the crude and adjusted models (OR: -0.56, 95% CI: -3.69-2.57, P: 0.72) (OR: -4.17, 95% CI: -9.28-0.94, P: 0.11), although this interaction was not statistically significant.

**Conclusion:**

According to the above findings, adherence to unhealthy food intake pattern increases odds of depression in MC4R risk allele (C allele) carriers. To confirm these findings, more studies are needed in the form of clinical trials and prospective studies with higher sample sizes.

## Introduction

Obesity and overweight are influential factors in today’s world, such that the prevalence of overweight and obesity in low-income countries, as well as in low-income groups in high-income countries, has steadily increased over the past century. In 2016, worldwide, 13% of adults over the age of 18 were reported to be obese and 39% to be overweight [1, 2]. Obesity, as a metabolic disease, is a major risk factor for other metabolic diseases such as coronary heart disease, ischemic stroke, and type 2 diabetes, and the physiological and psychological consequences of obesity, causes greater burden on health systems by disrupting people’s quality of life [3–5]. In the etiology of obesity, factors such as genetics [6], lifestyle [7], gender [8], diet [9], etc. are influential.

Mental disorders are a major public health problem in the world. In general, a mental disorder is a behavioral or mental pattern that causes discomfort or dysfunction in a person, and is significantly associated with decreased quality and life expectancy [10]. In 2020, about 150 million people worldwide were reportedly suffering from depression [11, 12]. Thus, it is predicted that after cardiovascular diseases, depression and anxiety will become the second leading risk of death in the world [13, 14]. Regarding the etiology of depression, factors such as genetics [15], lifestyle [16], gender [17], obesity [18], diet [16], etc. are influential. There are several studies on the relationship between diet, obesity, and depression [19–22], indicating that dietary patterns may have influence on the onset of depression [23–25]. Lifestyle changes in recent decades have led to changes in the consumption of food groups and the consumption of unhealthy foods, in addition to the direct impact on obesity [26, 27], has also been linked to depression [28]. Possible mechanisms of this association are increased inflammatory responses to overconsumption of unhealthy foods, due to the presence of chemical additives or due to dietary composition [29–31], as well as metabolic, physiological, and hormonal changes caused by excessive consumption of this style of dietary pattern [32–34]. Meanwhile, the consumption of healthy pattern diets, such as the Mediterranean diet, has been shown to have a protective effect on depression [35–37].

Variation in the MC4R gene variants, which are responsible for encoding the melanocortin 4 receptor, is the most common genetic cause of human obesity [38], and obesity itself is independently associated with depression [39]. The common Single nucleotide polymorphism (SNP) rs17782313 gene, close to MC4R, may also be significantly associated with higher total energy intake and dietary fat, thereby exerting an effect on dietary change [40]. Contemporary data suggests that synergistic interactions between environmental factors to dietary patterns may play an important role in pathogenesis of obesity and depression [6, 41]. In general, studies on the effect of the MC4R gene and depression are very limited. In a previous study, in addition to the association of this gene with obesity and the association of rs17782313 C allele variant with BMI, the association of this gene with depressed mood and overeating behaviors was reported [42]. In another study, the interactions of MC4R polymorphism (rs12970134) and dietary factors on metabolic syndrome were investigated, with the incumbent findings suggesting that there is an interaction between rs12970134 and Western diet, fat, and vegetable consumption on the risk of MetS or its components. In addition, there was also a significant correlation between rs12970134 and total fat and iron intake on the risk of abdominal obesity [43]. In a study that examined the interaction of MC4R variants and dietary patterns on the risk of obesity in Korean middle-aged adults, it was reported that the MC4R rs18882313 minor allele was more common in the obese group and that processed foods and fats (as a percentage of energy) was significantly higher, whilst fruit consumption was significantly lower, in people with MC4R minor alleles [44]. Moreover, it was shown that the interaction of mental stress interactions and energy intake with the MC4R minor allele genotype may increase the risk of obesity in Korean adults [44].

Most recent studies have shown that dietary patterns are associated with depression, and the association between obesity and overweight has been independently studied in the development of depression. However, since studies on the association between the MC4R gene and depression are very limited, and there is no study on the interaction of the MC4R gene with dominant dietary patterns and depression, this study, for the first time, sought to investigate the interaction of the MC4R gene and dietary patterns on depression.

## Materials and methods

### Study population

Two hundred and eighty-nine adult overweight (n = 136) and obese (n = 153) women from Tehran, Iran were enrolled in this cross-sectional study. Moreover, the study only included participants with good general health as self-certified by them. A mean age of 36.52 years (± 8.32 years) was determined from the range of 18 to 50 years. Subjects’ BMI ranged from 25 to 40.70 kg/m^2^. Blood samples and anthropometric measurements were taken at Tehran University of Medical Sciences (TUMS). An informed consent form was filled out by each participant before the study began. Hypertension, cardiovascular disease (CVD), diabetes mellitus, impaired liver renal function, use of medicine regularly, such as an oral contraceptive pill, smoking, excessive alcohol consumption, pregnancy, lactation, and menopause were excluded from the study. Furthermore, we excluded participants if they had a chronic illness that affected their diet, ate a special diet arbitrarily, or if they fluctuated in weight during the past year, or if their daily calories were less than 800 kcal and more than 4,200 kcal. The Ethics Commission of the TUMS granted ethical approval and assigned the number IR.TUMS.VCR.REC.1398.619.

### Assessment of dietary intake

In this study, a semi-quantitative FFQ of 147 questions was used to assess the dietary intake of individuals, which was administered by a trained nutritionist. FFQ was based on standard-sized foods commonly consumed by Iranians. Then, according to the participants’ reports, we collected the frequency of consumption of each meal of each food item that was consumed daily (e.g., bread), weekly (e.g., rice, meat), or monthly (e.g., fish) in the previous year. Also, the frequency reported for each food item was then converted into daily intake and the share of food consumed was converted to grams using household criteria [45]. Total energy intake was calculated by summing up energy intakes from all foods. The Iranian Food Composition Table [46] was used to determine the nutrient composition of Iranian food using United States Department of Agriculture (USDA) Food Composition data [47]. To identify the main patterns of diet, 147 nutrients were classified into 31 food groups. By using factor analysis based on eigenvalues larger than 1.5, two significant dietary patterns—later referred to as the HDP and the UDP—were identified. The principle component analysis (PCA) was carried out in order to separate these two eating patterns based on the 31 food groups. In the healthy dietary pattern, the consumption of food items included; vegetables, tomatoes, fruits, garlic, starchy vegetables, fruits, nuts, fish, bananas, and legumes. In the unhealthy dietary pattern, people were more likely to consume red meat, high-energy drinks, chicken, nuts, processed foods, high-fat dairy, low-cereals, low-spices, and less fruit.

### Assessment of depression

The 21-item DASS questionnaire was used to determine the status of depression. This questionnaire has two forms, short and long, each of which has 21 and 42 questions, respectively. The short form, introduced by Lovibond and Lovibond [48], has 21 terms that evaluates each of the psychological structures of depression, anxiety, and stress with 7 different term in which the validity of this tool in Iran has been confirmed [49]. Participants reported their responses to the items on this questionnaire on a 4-point Likert scale from 0 (does not apply to me at all) to 3 (applies to me most or most of the time). For each scale, scores were added for the specified items, and because DASS 21 is a short version of DASS (the long form has 42 items), finally, the total score of 21 questions is multiplied by 2, and for the depression subgroup, the score range between 10 and 13, 14–20, 21–27 and above 28 indicates mild, moderate, severe and very severe depression.

### DNA extraction and sequencing of the gene

The MC4R gene primer was selected based on a previous study [50]. According to the manufacturer’s protocol, we extracted genomic DNA from blood samples with the use of the Mini Columns, Type G kit (GeneALL, Exgene). In addition, with the use of a Nano Drop spectrophotometer (Thermo Scientific Company, USA), we measured the concentration and purity of extracted DNA. We stored the extracted DNA at 4ºC, before sequencing was performed. The polymerase chain reaction (PCR) was performed using the following primers: forward primer 5-AAGTTCTACCTACCATGTTCTTGG-3 and reverse primer 5- TTCCCCCTGAAGCTTTTCTTGTCATTTTGAT-3. PCR reactions were performed in a final volume of 20 *µ*l, containing 1 *µ*l extracted DNA, 0.5 *µ*l primers F, 0.5 *µ*l primers R, 10 *µ*l Permix (Amplicon, Germany), and 8 *µ*l Distilled water, with the following conditions in a DNA thermocycler: 1- primary denaturation at 95 °C for 2 min; 2- Thirty- five cycles of denaturation at 95 °C for 30 s, annealing at 58 °C for 30 s, extension at 72 °C for 30 s; 3- final extension at 72 °C for 5 min; 4- final step at 4 °C. Amplified DNA (7 *µ*l) was digested with 0.5 *µ*l of BCII restriction enzyme (Fermentase, Germany) at 56 °C overnight. All products were visualized by agarose gel electrophoresis. Then, fragments containing three genotypes were distinguished: CC, CT, and TT [51].

### Biochemical assessments

Fasting serum glucose (FSB), insulin, total cholesterol (TC), triglyceride (TG), low density lipoprotein (LDL), and high-density lipoprotein (HDL) was measured from blood samples drawn after 8–12 h of overnight fasting. All samples were assessed by standard methods at the Nutrition and Biochemistry Laboratory of the school of Nutritional and Dietetics at Tehran University of medical sciences.

### Anthropometric assessments

Weight was measured with digital scales, to the nearest 100 g, with minimal clothing and no shoes. Participants’ height was measured with a stadiometer, in a standing position and without shoes. BMI was calculated by dividing weight (kg) by height squared (cm), and neck circumference (NC) was measured below the laryngeal ridge and perpendicular to the long axis of the neck [52], and the minimum circumference was recorded with the nearest 0.1 cm.

### Body composition analysis

Body composition was measured using a multi-frequency Bioelectrical Impedance Analyzer (BIA), InBody 770 scanner (Inbody Co., Seoul, Korea), which records weight, body mass index (BMI), Body Fat Mass (BFM), Fat-free Mass (FFM), Waist Circumference (WC), and Hip Circumference (HC). According to the manufacturer’s instructions, first, participants removed their shoes, coats, and sweaters, then they stood on the balance scale, barefooted, and held the handles of the machine.

### Assessment of other variables

Levels of physical activity, using a valid questionnaire (International Physical Activity Questionnaire - short form (IPAC)), which includes all areas of physical activity (occupation, transportation, yard / garden, home and leisure) and sitting and as a metabolic equivalent Hours per week (MET-h / wk), were evaluated [53]. The questionnaire included questions in 5 areas of activity: job-related physical activity; Physical transport activity; Activities for housework and house maintenance; Recreation, exercise and physical activity in leisure time; And the time spent sitting the participant’s pants were asked to think about all the intense and moderate activity they had done in the past 7 days, taking into account the time spent on these activities. It was classified as: low < 600 (MET-h / wk), moderate = 600–3500 (MET-h / wk) and severe > 3500 (MET-h / wk)[54]. A demographic questionnaire was used to collect information on age, marital status, and family history of obesity and overweight, which was defined as BMI ≥ 30 kg/m^2^ and 25 ≤ BMI ≤ 29.9 kg/m^2^, respectively [55].

### Statistical analysis

The distribution of data was investigated using the Kolmogorov-Smirnov test. Quantitative data were reported as mean and standard deviation and qualitative data were expressed as number and percentage, respectively. We used principal component analysis (PCA) to identify the main dietary patterns based on 31 food groups, based on scree plot inspection and eigenvalues over 1.5. Varimax rotation was used to achieve a simple matrix with better ability to interpret and extract dominant dietary patterns. According to previous studies, and because of the nature of the data and correlations, values with a load factor greater than 0.58 were considered to determine the items of each food pattern. Factor loads illustrate correlation coefficients between food groups and dietary patterns, and a positive charge in a factor indicates a direct relationship with the agent, while a negative charge indicates that the food is inversely related to the agent. Dietary patterns were labeled based on the researchers’ interpretation of the data, and participants were classified based on the mean scores of healthy, unhealthy, which resulted in low and high intakes. Independent t-tests were performed to assess differences in participants’ general characteristics (such as age, anthropometry, and physical activity) in the dietary patterns and depression. The distribution of qualitative variables in the groups was assessed using Chi-square test. To determine the relationship between main dietary patterns and MC4R gene and depression, logistic binary regression was performed in crude and adjusted models. Adjustments were made for age, economic status, night sleep status, a history of weight loss, and physical activity. In all multivariate models, the low adherence of dietary patterns score was considered as reference. Comparison of quantitative variables between quartiles of dietary pattern or genotype was performed using one-way analysis of variance (ANOVA) and analysis of covariance (ANCOVA). The interaction between dietary pattern and genotype on quantitative variables was assessed using linear regression model analysis. All statistical analyses were performed using SPSS software (version 23; SPSS Inc, Chicago IL), with statistical significance accepted, a priori, at P < 0.05.

## Result

### Study population characteristics

In this cross-sectional study, the demographic characteristics of individuals are reported in Table 1. The means and standard deviation (SD) of age, weight, and BMI of individuals were 36.52 (8.32) years, 78.75 (11.51) kg, and 30.33 (3.65) kg/m ^2^, respectively. The frequencies of T and C alleles of rs17782313 were 41.8% and 58.2%. The overall prevalence of rs17782313 genotype was 29.4%, 24.8%, and 45.7% for TT, TC, and CC, respectively. Among the participants in terms of marital status, 27.3% were single and 72.6% were married. The majority of study population (99.3%) were employed, and 22.1% were economically weak, 44.2% were moderate, and 33.5% had good economic condition. Among the participants in terms of depression, 53.2% were normal, 13.3% were mild, 17.9% were moderate, 9.3% were severe, and 6.1% were very severe.

**Table 1:** Characteristics of study participants (n = 289)

Variables		Mean ± SD	Minimum	Maximum
**Demographic assessments**				
Age (year)		36.5 ± 8.3	18	50
PA (MET-minutes/week)		996.3 ± 1077.5	66	7296
**Biochemical assessments**				
Total Cholesterol (mg/dl)		185.6 ± 38.5	104	433
TG (g/dl)		118.4 ± 64.5	37	512
LDL (mg/dl)		97 ± 26.9	34	282
HDL (mg/dl)		47.8 ± 10.9	18	84
FBS		87.7 ± 9.7	71	137
**Body composition**				
WC (cm)		98.4 ± 9.2	80.1	123.2
HC (cm)		113.4 ± 13	63	160
BFM (kg)		34 ± 8.7	19.4	74.2
FFM (kg)		46.8 ± 5.6	35.3	67.7
Height (cm)		161.2 ± 5.9	142	179
Body weight (kg)		78.7 ± 11.5	57.7	119.5
BMI (kg/m^2^)		30.3 ± 3.6	25	40.7
**Mental health parameter**				
Depression score		10.6 ± 9.4	0	40
**Qualitative variables**				
Economic status	Low level	64	22.1	
	Moderate level	128	44.2	
	High level	97	33.5	
Job status	unemployed	2	0.6	
	employed	287	99.3	
Marital status	Single	79	27.3	
	Married	210	72.6	
Education status	Illiterate	3	0.3	
	under diploma	37	12.8	
	Diploma	106	36.6	
	bachelor and higher	143	49.4	

SD: Standard deviation; BMI: Body mass index; TG: Triglyceride; LDL: Low density lipoprotein; HDL; High density lipoprotein; PA: physical activity; BFM: body fat mas; FFM: fat free mass; WC: waist circumference; HC: Hip Circumference

Quantitative variables with mean and SD and qualitative means with number and percentage have been reported.

### Association between biochemical parameters, body composition among rs17782313 genotype

The general characteristics of the participants among the MC4R genotype are presented in Table 2. A total of 289 Iranian women were categorized based on rs17782313 genotype, and divided into three groups: TT genotype (n = 96), TC genotype (n = 96), and CC genotype (n = 97). Although there was a significant marginal difference in educational status (P = 0.08), among other variables, no significant relationship was observed with MC4R gene variants, even after adjustment for BMI, age, total energy intake, and physical activity (P > 0.05).

**Table 2:** Characteristics of participants among MC4R gene genotypes (n = 289)

Variables		TT (n = 96)	TC (n = 96)	CC (n = 97)	P value*	P value**
**Demographic assessments**						
Age (year)		37.4 ± 7.8	37.0 ± 9.6	35.8 ± 8	0.33	0.23
PA (MET-minutes/week)		1081 ± 1140.2	1422.2 ± 2497.8	1177.1 ± 2371.6	0.63	0.58
**Biochemical assessments**						
Total Cholesterol (mg/dl)		183.3 ± 34	187.5 ± 34.3	184.8 ± 38.1	0.79	0.60
TG (g/dl)		0.3 ± 0.5	0.38 ± 0.5	0.3 ± 0.4	0.40	0.68
LDL (mg/dl)		92.2 ± 22.3	100.2 ± 23.7	93.8 ± 25.2	0.12	0.19
HDL (mg/dl)		45.5 ± 9.7	47.4 ± 10.1	47.1 ± 12	0.50	0.38
FBS		86.7 ± 8.9	89.4 ± 11.6	86.2 ± 8.5	0.09	0.25
**Body composition**						
WC (cm)		93.6 ± 17.3	96.3 ± 10.8	96 ± 17.7	0.63	0.43
HC (cm)		114.7 ± 10.6	114 ± 11.5	113.4 ± 7.7	0.76	0.42
BFM (kg)		34.5 ± 9.7	33.4 ± 8.1	33.7 ± 8.3	0.69	0.84
FFM (kg)		47.6 ± 5.8	46.9 ± 5.2	46.2 ± 5.6	0.21	0.16
Height (cm)		162.1 ± 5.9	161.7 ± 5.8	161 ± 5.8	0.39	0.15
Body weight (kg)		81.5 ± 13.1	80.5 ± 11.4	80.3 ± 12.5	0.77	0.53
BMI (kg/m^2^)		31.3 ± 4.9	30.8 ± 4.4	30.8 ± 3.90	0.71	0.72
**Mental health parameters**						
Depression score		5.1 ± 4.9	5.3 ± 4.4	5.5 ± 4.9	0.87	0.91
**Qualitative variables**						
Economic status	Low level	29(10)	17(5.8)	18(6.2)	0.38	0.88
	Moderate level	40(13.8)	32(11)	56(19.3)		
	High level	27(9.3)	47(16.2)	23(7.9)		
Job status	unemployed	47(16.2)	55(19)	61(21.1)	0.66	0.21
	Occupying	49(16.9)	41(14.1)	36(12.4)		
Marital status	Single	23(7.9)	23(7.9)	19(6.5)	0.69	0.95
	Married	73(25.2)	73(25.2)	78(26.9)		
Education status	Illiterate	0	0	2(0.6)	0.10	0.08
	under diploma	8(2.7)	12(4.1)	14(4.8)		
	Diploma	33(11.4)	37(12.8)	38(13.4)		
	bachelor and higher	55(19)	47(16.2)	43(14.8)		

SD: Standard deviation; BMI: Body mass index; TG: Triglyceride; LDL: Low density lipoprotein; HDL; High-density lipoprotein; PA: physical activity; BFM: body fat mas; FFM: fat free mass; WC: waist circumference; HC: Hip Circumference.

Quantitative variables with mean and SD and qualitative means with number and percentage have been reported.

Data are indicated as Mean ± SD otherwise indicated Categorical variables have shown as n (%).

P value * was obtained from t-test and P value ** was obtained from ANOVA test.

The variables are adjusted to the BMI age, physical activity and energy intake.

### Association between dietary intakes among rs17782313 genotype

The food group and nutrient intakes according to MC4R rs17782313 genotype are shown in Table 3. The results of this study, after controlling for confounding factor (energy intake), shows that those with CC risk alleles consumed significantly less B carotene than those with TT and TC alleles. Among all micronutrients, only a significant correlation was shown with B carotene (P = 0.05), and this correlation remained significant after adjusting for energy intake (P = 0.03). Among other variables, no significant relationship was observed with MC4R gene varieties and dietary intake, even after adjusting the total energy intake (P > 0.05).

**Table 3:** Dietary intake in individuals with MC4R gene variant (rs17782313) (n = 289)

Variables	TT (n = 96)	TC (n = 96)	CC (n = 97)	P value*	P value**
**Macronutrients**					
Energy (Kcal)	2614.3 ± 753.5	2588.4 ± 709.5	2602.7 ± 732.9	0.77	
Protein (g/day)	89.9 ± 27.8	89.3 ± 27.8	86.8 ± 29	0.70	0.31
Carbohydrate (g/day)	368.7 ± 117.9	377.4 ± 130.8	367.7 ± 111.3	0.85	0.88
Total Fat (g/day)	91.7 ± 30	96 ± 34.2	94 ± 33.6	0.72	0.79
**Fat subtypes**					
Cholesterol (g/day)	254.6 ± 98.3	258.3 ± 113.6	244.2 ± 99.5	0.61	0.61
Saturated fatty acids (mg/day)	27.2 ± 9.8	29.3 ± 13.4	27.3 ± 9.9	0.39	0.49
MUFA (mg/day)	30.7 ± 10.2	31.1 ± 12.3	31.4 ± 12.4	0.91	0.75
PUFA (mg/day)	19.7 ± 8.1	19.2 ± 9.3	20.4 ± 9.6	0.66	0.37
Oleic acid (mg/day)	27.4 ± 9.7	28 ± 11.8	28.2 ± 11.8	0.86	0.76
Linoleic acid (mg/day)	16.9 ± 7.8	16.6 ± 8.8	17.7 ± 9.1	0.66	0.42
Linolenic acid (mg/day)	1.2 ± 0.6	1.2 ± 0.7	1.2 ± 0.7	0.84	0.66
EPA (mg/day)	0.03 ± 0.03	0.03 ± 0.04	0.03 ± 0.03	0.69	0.74
DHA (mg/day)	0.1 ± 0.1	0.1 ± 0.1	0.1 ± 0.1	0.76	0.82
Trans fatty acid (mg/day)	0.00 ± 0.00	0.00 ± 0.00	0.00 ± 0.00	0.86	0.85
**Fat-soluble vitamins**					
Vitamin A (mg/day)	842.4 ± 445.7	755.5 ± 428.3	734.1 ± 352	0.15	0.07
β-carotene (mg/day)	6046 ± 4215.6^a^	5112.3 ± 3348.6	4828.2 ± 3111.7^a^	**0.05**	**0.03**
α-carotene (mg/day)	1265.5 ± 1007.9	1077.6 ± 1290.6	1058.9 ± 974.0	0.36	0.31
Lutein (mg/day)	2578.0 ± 1802.8	2226.9 ± 1433.6	2183.4 ± 1982.2	0.27	0.21
β-Cryptoxanthin (mg/day)	376.3 ± 293.1	342.7 ± 231.1	335.8 ± 239.6	0.51	0.35
Lycopene (mg/day)	7222.7 ± 5316.2	6611 ± 5038.8	6155.6 ± 4632.6	0.31	0.24
Vitamin k (mcg/day)	249.9 ± 271.1	204 ± 103.3	197.7 ± 170.9	0.14	0.12
Vitamin D (µg/day)	1.9 ± 1.4	2 ± 2	1.94 ± 1.5	0.94	0.98
Vitamin E (mg/day)	17.4 ± 9.3	16.3 ± 7.5	17.44 ± 9.63	0.66	0.47
α.tocopherol	11.7 ± 6.2	10.8 ± 4.8	11.5 ± 6.2	0.60	0.41
**Water soluble vitamins**					
Vitamin C (mg/day)	204.8 ± 119.9	186.5 ± 106.4	190.2 ± 138.7	0.61	0.35
Thiamin (mg/day)	2 ± 0.6	2.1 ± 0.7	2 ± 0.6	0.34	0.26
Riboflavin (mg/day)	2.2 ± 0.7	2.2 ± 0.8	2.2 ± 0.8	0.94	0.78
Niacin (mg/day)	25.2 ± 8.7	25.4 ± 8.1	25 ± 10.1	0.96	0.85
Pantothenic acid (mg/day)	6.5 ± 1.9	6.5 ± 2.3	6.4 ± 2.7	0.95	0.79
Vitamin B6 (mg/day)	2.2 ± 0.7	2.2 ± 0.7	2.1 ± 0.7	0.57	0.15
Biotin (mcg/day)	39.9 ± 13.7	36.5 ± 15.1	37.8 ± 19.7	0.45	0.15
Folate (µg/day)	613.6 ± 162.6	614.7 ± 199.3	591.8 ± 169.8	0.57	0.27
Vitamin B12 (mcg/day)	4.3 ± 2.3	4.3 ± 2.1	4.3 ± 2.2	0.99	0.87
**Minerals**					
Calcium (mg/day)	1180.1 ± 401.9	1179.9 ± 446.6	1126.8 ± 394.4	0.56	0.40
Iron (mcg/day)	18.5 ± 5.7	18.8 ± 6.2	18.4 ± 5.8	0.88	0.76
Phosphor (mg/day)	1659.7 ± 492.5	1654 ± 556.9	1594.4 ± 495	0.59	0.24
Magnesium (mg/day)	469.8 ± 145.1	464.9 ± 164.6	443.3 ± 135.6	0.38	0.06
Zinc (mg/day)	12.9 ± 3.8	13.2 ± 4.6	12.7 ± 4.1	0.72	0.74
Copper (mcg/day)	2 ± 0.6	1.9 ± 0.7	2 ± 0.8	0.72	0.10
Manganese (mg/day)	7.2 ± 3.2	7.5 ± 3.3	6.7 ± 2.2	0.18	0.15
Selenium (mcg/day)	120.3 ± 41.1	125.4 ± 50.8	115.4 ± 38.4	0.28	0.21
Floride (mg/day)	2783.5 ± 3729.2	2887.9 ± 2203.7	2417.2 ± 1658	0.40	0.42
Chromium (mg/day)	0.1 ± 0.1	0.1 ± 0.1	0.1 ± 0.1	0.28	0.32
Sodium (mg/day)	4149.2 ± 1325.8	4451.6 ± 1386.3	4169.7 ± 1485.9	0.33	0.42
Potassium (mg/day)	4470.9 ± 1542.6	4281 ± 1653.6	4220.2 ± 1494.9	0.51	0.10

MUFA: Monounsaturated fatty acids; PUFA: Polyunsaturated fatty acids; EPA: Eicosapentaenoic acid; DHA: Docosahexaenoic acid

Data are indicated as Mean ± SD. P value * was obtained from t-test and P value ** was obtained from ANOVA test. The variables are adjusted to the energy intake.

### Factor loadings of food groups in identified dietary patterns

**Fig. 1:**
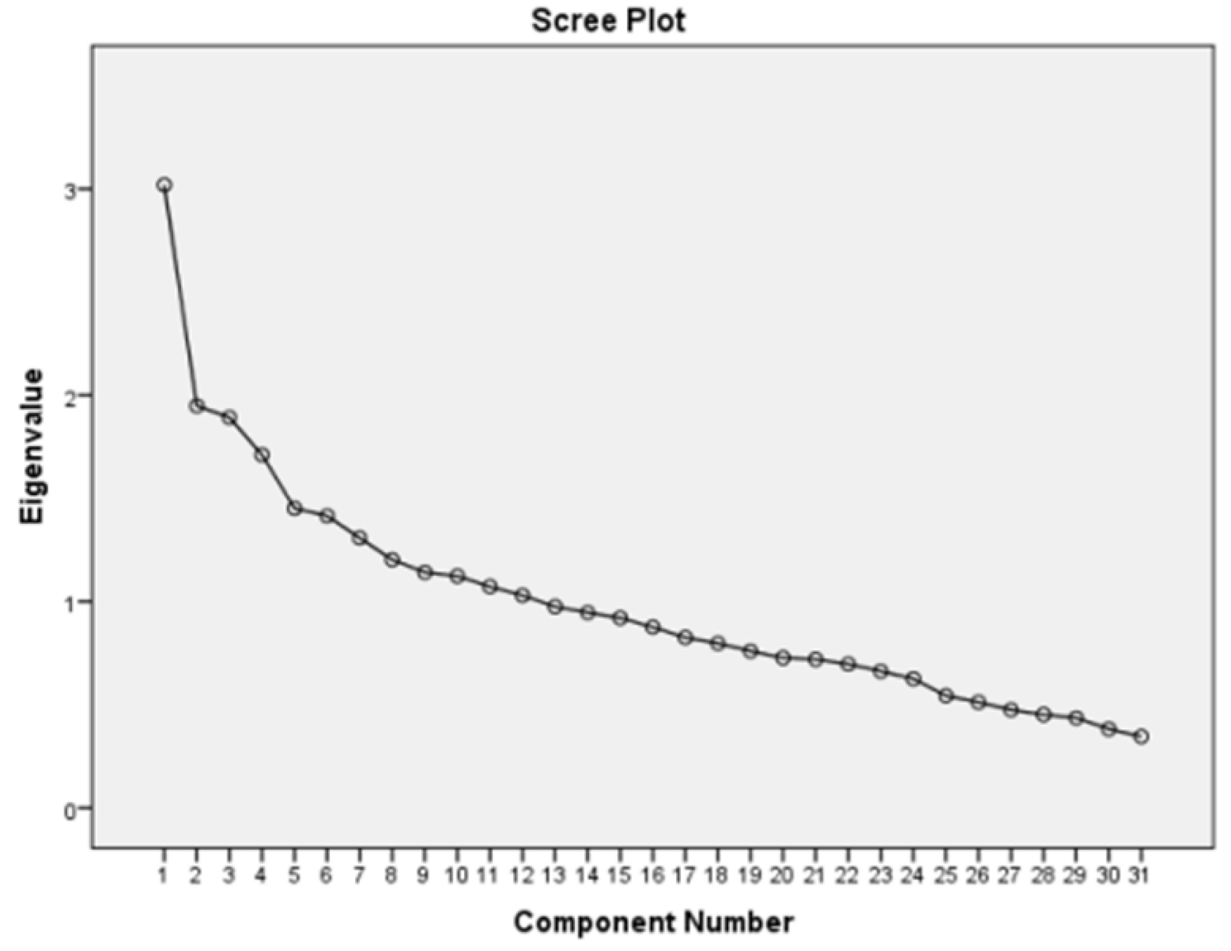
Scree plot of the nutrients and the extracted principal components in overweight and obese women

Table 4 shows the factor loadings of food groups in identified dietary patterns. Nutrient patterns were derived using PCA with varimax rotation and based on the correlation matrix. Factor load values less than 0.2 have been omitted and the Kaiser—Meyer—Olkin measure of sampling adequacy (KMO) was 0.558. 31 Food group were selected for factor analysis, including vegetables, tomato, low fat dairy, nuts ,garlic ,starchy, fruit, walnut ,fish, banana, red Meat, high energy drink, chicken, nuts ,processed food, high-fat dairy, beans, grain and spices. Factor scores for all participants for each of the extracted factors were calculated by summing the frequency of consumption, multiplied by factor loadings across food groups. By using the factor analysis method and according to the Scree Plot chart review, two dominant dietary patterns were identified in the subjects (Fig. 1). Patterns were named based on food groups as the healthy pattern and unhealthy pattern. The healthy eating pattern included vegetables, tomatoes, low-fat dairy, nuts, garlic, starchy vegetables, fruits, walnuts, fish, bananas, dried red meat, cereals, spices, and legumes (percentage variance = 9.73). The UDP included red meat, high energy drinks, chicken, processed foods, and high fat dairy (percentage of variance = 6.28).

**Table 4:** Factor loadings of the food groups in the main dietary patterns extracted (n = 289)

Food groups	HDP	UDP
**Vegetables**	0.701	-
**Tomato**	0.632	-
**Low fat dairy**	0.459	-
**Nuts**	0.441	-0.336
**Garlic**	0.422	-
**Starchy vegetables**	0.402	-
**Fruit**	0.390	-
**Walnut**	0.374	-
**Fish**	0.340	-
**Banana**	0.307	-
**Red Meat**	0.366	0.410
**High energy drink**	-	0.396
**Chicken**	-	0.395
**Nuts**	-	-0.378
**Processed food**	-	0.371
**High-fat dairy**	-	0.356
**Beans**	0.348	-0.349
**Grain**	-	-0.307
**Spices**	-	-0.301

HDP: healthy dietary pattern; UDP: unhealthy dietary pattern.

Percentage variance of healthy dietary pattern = 9.73

Percentage variance of unhealthy dietary pattern = 6.28

Cumulative variance percentage = 16

### Association between biochemical parameters, body composition among adherence of dietary patterns

The general characteristics of the participants among the food groups extracted are presented in Table 5. All participants were divided into two groups based on the healthy and unhealthy food patterns extracted. For the healthy dietary pattern, there was a significant difference in height (P = 0.01), FBS (P = 0.05) and FFM (P = 0.01) in the crude model. After adjusting BMI, age, total energy intake and physical activity, Body weight (P = 0.04), Job (P = 0.03) and educational status (P < 0.001) showed a significant difference. In this regard, the economic status (P = 0.004) maintained its significance after adjusting for confounding factors. There was no significant difference for the other variables in Table 5 compared to the HDP (P > 0.05). But, in terms of following an unhealthy dietary pattern, in the crude model, there was a significant difference with height (P = 0.009), Economic (P = 0.01) and marital status (P = 0.03). After adjusting for BMI, age, total energy intake, and physical activity, a significant difference was observed between groups for HC (P = 0.03). In this regard, Age (P < 0.001) and physical activity (P = 0.01) remained significant after controlling for potential cofounders.

**Table 5:** Characteristics of participants among dietary patterns (n = 289)

		Healthy pattern				Unhealthy pattern			
**Variables**		Low intake (n = 144)	High intake (n = 145)	P value*	P value**	Low intake (n = 145)	High intake (n = 144)	P value*	P value**
**Demographic assessments**									
Age (year)		36.2 ± 8.7	36.8 ± 8.3	0.51	0.10	38.4 ± 7.8	34.6 ± 8.7	**< 0.001**	**< 0.001**
PA (MET-minutes/week)		1010.2 ± 2102.8	1403.1 ± 2094.5	0.13	0.13	1462.6 ± 2773.8	928.6 ± 941.5	**0.04**	**0.01**
**Biochemical assessments**									
Total Cholesterol (mg/dl)		184.4 ± 38	185.9 ± 34.5	0.73	0.41	187.8 ± 34.9	182.2 ± 37.6	0.23	0.47
TG (g/dl)		0.3 ± 0.5	0.34 ± 0.5	0.86	0.52	0.35 ± 0.5	0.34 ± 0.5	0.86	0.66
LDL (mg/dl)		94.5 ± 25.3	95.6 ± 23.1	0.72	0.06	97.8 ± 23.4	92 ± 24.8	0.06	0.32
HDL (mg/dl)		46.9 ± 10.5	46.7 ± 11.2	0.84	0.77	46.8 ± 11.2	46.8 ± 10.5	0.96	0.46
FBS		88.7 ± 11	86.3 ± 7.9	**0.05**	0.06	87.6 ± 10.4	87.4 ± 8.7	0.86	0.40
**Body composition**									
WC (cm)		93.9 ± 16.4	97.2 ± 15.4	0.18	0.51	95 ± 18.7	96.3 ± 11.7	0.59	0.91
HC (cm)		113.7 ± 9.9	114.6 ± 9.7	0.56	0.65	113.5 ± 8.7	115 ± 11	0.31	**0.03**
BFM (kg)		33.8 ± 8.4	34.2 ± 8.9	0.65	0.93	33.2 ± 7.7	34.7 ± 9.5	0.14	0.06
FFM (kg)		46 ± 5.4	47.6 ± 5.6	**0.01**	0.06	46.5 ± 5.7	47.1 ± 5.5	0.35	0.47
Height (cm)		160.4 ± 6	162.8 ± 5.7	**0.01**	0.13	160.4 ± 6.2	162.2 ± 5.5	**< 0.001**	0.67
Body weight (kg)		79.6 ± 11.6	81.8 ± 12.8	0.14	**0.04**	79.8 ± 11.1	81.6 ± 13.2	0.21	0.95
BMI (kg/m^2^)		31 ± 4.3	31 ± 4.3	0.99	0.39	31 ± 4	31.1 ± 4.6	0.86	0.58
**Mental health parameters**									
Depression score		5.5 ± 4.9	5 ± 4.5	0.42	0.27	5.2 ± 4.8	5.3 ± 4.6	0.79	0.15
**Qualitative variables**									
Economic status	Low level	39(13.4)	40(13.8)	**0.03**	**< 0.001**	49(16.9)	31(10.7)	**0.01**	0.20
	Moderate level	78(26.9)	61(21.1)			56(19.3)	81(28)		
	High level	27(9.3)	44(15.2)			40(13.8)	32(11)		
Job status	unemployed	90(31.1)	85(29.4)	0.59	**0.03**	85(29.4)	90(31.1)	0.13	0.81
	Occupying	54(18.6)	60(20.7)			60(20.7)	54(18.6)		
Marital status	Single	29(10)	35(12.1)	0.42	0.11	24(8.3)	40(13.8)	**0.03**	0.74
	Married	115(39.7)	110(38)			121(41.8)	104(35.9)		
Education status	Illiterate	3(1)	0	0.08	**< 0.001**	2(0.6)	1(0.3)	0.77	0.29
	under diploma	25(8.6)	15(5.1)			23(7.9)	17(5.8)		
	Diploma	45(15.5)	41(14.1)			43(14.8)	43(14.8)		
	bachelor and higher	71(24.5)	89(30.7)			77(26.6)	83(28.7)		

SD: Standard deviation; BMI: Body mass index; TG: Triglyceride; LDL: Low density lipoprotein; HDL; High-density lipoprotein; PA: physical activity; BFM: body fat mas; FFM: fat free mass; WC: waist circumference; HC: Hip Circumference.

Quantitative variables with mean and SD and qualitative means with number and percentage have been reported.

P value * was obtained from t-test and P value ** was obtained from ANOVA test.

The variables are adjusted to the BMI age, physical activity and energy intake.

### Interaction between dietary patterns and MC4R gene variants on depression

**Fig. 2:**
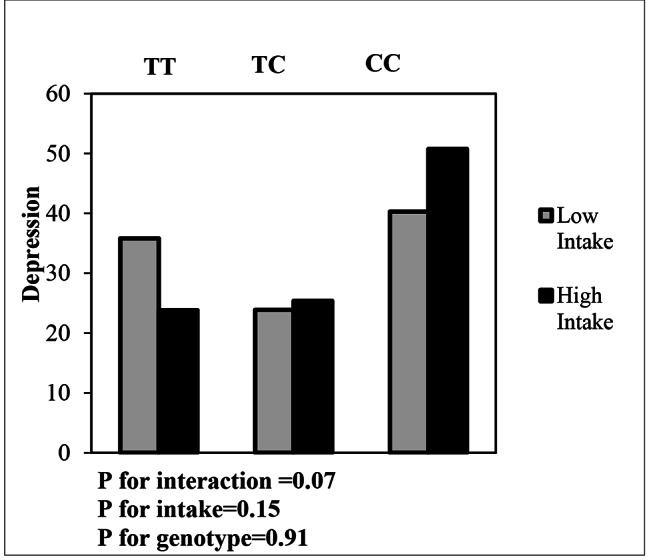
Interaction between MC4R genotypes (TT as the reference group) and UDP on depression (The *P* value for CC genotype: 0.91; *P* value for UDP: 0.15; *P* value for interaction between CC genotype and UDP: 0.07)

**Fig. 3:**
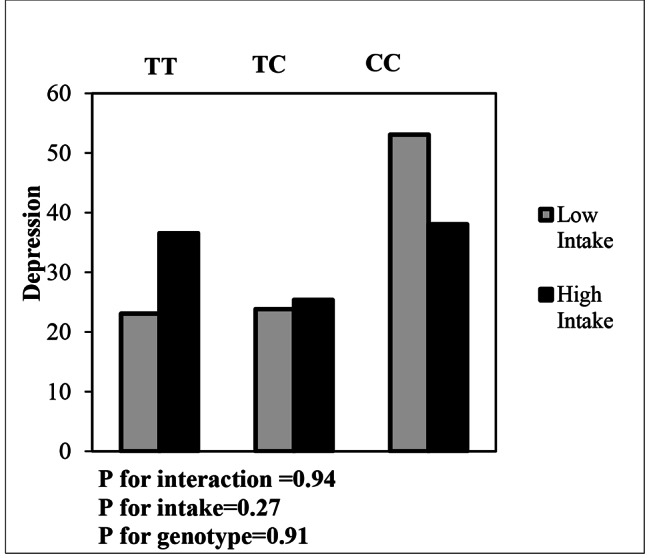
Interaction between MC4R genotypes (TT as the reference group) and HDP on depression (The *P* value for CC genotype: 0.91; *P* value for HDP: 0.277; *P* value for interaction between CC genotype and HDP: 0.94)

Interaction between dietary patterns and MC4R gene variants and the effect on depression is shown in Table 6. Using the Generalized linear Model (GLM), the interaction between MC4R polymorphism (rs17782313) and dietary patterns on depression was examined. No Significant interactions were observed between UDP score and rs17782313 SNP in the crude model for people with CC (OR: 0.60, 95%CI: -1.22-2.43, P: 0.51) and CT (OR: 0.95, 95%CI: -1.14-3.06, P: 0.37) genotypes on depression. In adjusted model, participants with the CC genotype, who had the highest adherence to the UPD score, were more likely to develop depression, after controlling for confounders (age, economic status, Night sleep status, a history of weight loss and physical activity) (OR: 8.77, 95%CI: -0.86-18.40, P: 0.07) (Fig. 2). Although the interaction of CT genotype with UDP in the crude model was not significant on depression (OR: 0.95, 95%CI: -1.14-3.06, P: 0.37), a positive logical relationship with depression in was observed adjusted model (OR: 3.78, 95% CI: -5.88-13.45, P: 0.44), after controlling for confounding factors. Regarding the interaction of MC4R gene and HDP on depression (Fig. 3), logical inverse relationships were observed between CT genotype and HDP on depression in both crude and adjusted models (OR: -0.56, 95% CI: -3.69-2.57, P: 0.72) (OR: -4.17, 95% CI: -9.28-0.94, P: 0.11), although this interaction was not statistically significant in both models. In individuals with CC genotype, there was no significant relationship between depression and increased adherence to a HDP in both models.

**Table 6:** Interaction of MC4R gene (rs17782313) and healthy and unhealthy dietary patterns on depression in obese and overweight women (n = 289)

	Healthy pattern			Unhealthy pattern		
Depression	High intake			High intake		
Crude	OR	95% CI	P-value	OR	95% CI	P-value
CC	0.96	-1.8-3.7	0.48	0.60	-1.2-2.4	0.51
CT	-0.56	-3.7-2.6	0.72	0.95	-1.1-3.1	0.37
TT	Reference					
**Model 1**						
CC	0.17	-4.4-4.7	0.94	8.77	-0.9-18.4	**0.07**
CT	-4.17	-9.3-0.9	0.11	3.78	-5.9-13.4	0.44
TT	Reference					

OR: odds ratio, CI: confidence interval

P-values < 0.05 were considered as significant and 0.05, 0.06 and 0.07 were considered as marginal significant.

Low intake is considered as a reference group.

Model 1 is considered as an adjusted model

The variables are adjusted to the age, economic status, Night sleep status, history of weight loss and physical activity.

## Discussion

The aim of this study was to determine the relationship between dominant dietary patterns and MC4R with depression in overweight and obese Iranian adults. After analyzing the variables of dominant dietary patterns by method, unhealthy and healthy dietary patterns were obtained. In the unhealthy dietary pattern, consumption of food items, such as eat red meat, high-energy drinks, chicken, nuts, processed foods, and high-fat dairy was higher. In contrast, in the healthy dietary pattern, people were more likely to consume vegetables, tomatoes, fruits, garlic, starchy vegetables, fruits, nuts, fish, bananas and legumes. The hypothesis of this study regarding the effect of dietary patterns on mental health was that greater adherence to a HDP would be associated with lower odds of developing depression. In this study, the relationship between dominant dietary patterns and MC4R with the likelihood of depression, after adjusting for the effects of confounders such as age, economic status, night sleep status, a history of weight loss, and physical activity, was seen with increasing adherence to unhealthy dietary pattern. In this investigation, in adjusted model, participants with the CC genotype who had the highest adherence to the UPD score were more likely to develop depression, but no significant relationship was observed in the crude model. On the other hand, in the present study, data analysis showed that HDP in this study was not significantly associated with the score of depression in crude and adjusted models. Also, the HDP was not significantly associated with the odds ratio of depression in the crude and adjusted models. However, the relationship between following a HDP and lower odds ratio of depression in the adjusted model in people with CT alleles was close to significant and in both models, where an inverse relationship with CT gene and consumption of HDP with depression was observed.

So far, various studies have been conducted pertaining to the relationship between diet and the incidence of depression. Kim et al. found that the “Western” dietary pattern, which consisted of refined grains, white potatoes, cheese, meat, oils and fats, and high sugar, was significantly associated with depression in both sexes. Moreover, a healthy dietary pattern, consisting whole grains, fruits, vegetables, fish, nuts, and seeds was inversely associated with depression (OR) in women [56]. A study by Maryam Khosravi et al., performed on 330 depressed patients (case) and healthy individuals (control) (1: 2), found two main dietary patterns using factor analysis. These patterns were an unhealthy dietary pattern, including high-refined grains and breads, high-fat dairy, solid oils, liquid oils and mayonnaise, pickles, snacks, soft drinks, fruits and industrial juices, red meat, poultry, processed meats, and sweets, in addition to a healthy dietary pattern, which included fruits, cruciferous vegetables, yellow, green leaves and other vegetables, low fat dairy products, whole grains, nuts, and olives. Accordingly, findings from this study suggest that healthy and unhealthy dietary patterns may be associated with a higher risk of depression [24]. A prospective study in 2011 revealed a significant dose-dependent and inverse relationship between PUFA and MUFA intake and depression, and participants with the highest trans-FA intake showed significantly higher risk of depression [57]. Furthermore, sugar consumption has also been linked to depression [58]. Possible mechanisms of unhealthy dietary patterns include the presence of more processed foods which are linked to an increased risk of cardiovascular disease, as well as increased inflammation[59, 60], both of which are involved in the pathogenesis of depression [61, 62]. In people with major depressive disorder, the level of proinflammatory cytokines increases, and cytokines can affect the incidence of depression by altering neurotransmitter metabolism, endocrine nerve function, and regional brain activity [63]. Dietary folate deficiency, on the other hand, can reduce access to S-adenosylmethionine, which is responsible for methyl transport in the body, thereby disrupting the formation of myelin, neurotransmitters, and membrane phospholipids by increasing the risk of depression [34].

There are several studies pertaining to the relationship between healthy dietary patterns and depression. For instance, a cohort study of 3,523 people in France found that greater adherence to the Mediterranean dietary pattern in mid-life was associated with a reduced risk of developing depressive symptoms, especially in men [64]. A study by Skarupski et al. also found that following a diet high in vegetables, fruits, whole grains, fish, and legumes could have a protective effect against the development of depressive symptoms at older ages [65]. In a study of 64 girls, aged 17 − 15 years with normal body mass index (BMI), the relationship between walking exercise and banana consumption was examined for depressive symptoms, where banana consumption, alone and with exercise, was significantly associated with lower depression score than the control group [66].

Previous studies in this field have been limited to investigating the relationship between the dominant food intake pattern and mental disorders [25, 67–70]. They have also evaluated the interaction of MC4R and different diets on different outcomes (metabolic syndrome, diabetes and obesity) [71, 72]. According to a contemporary approach to nutrition epidemiology, where the role of mediators between diet and genes is considered, we, for the first time in Iran and the world, discerned the interaction between the dominant food intake pattern and MC4R 17782313rs on mood disorders (depression). A study examining the interaction of MC4R rs17782313 with mental stress and energy intake and the risk of obesity found that interactions of mental stress and energy intake with the MC4R minor allele genotype may be associated with an increased risk of obesity in Korean adults [44]. Another study showed that the relationship between MC4R 17782313rs polymorphism and type 2 diabetes depends on diet. In fact, people with a C-risk allele had a lower risk of type 2 diabetes with less adherence to the Mediterranean dietary pattern, whilst people with a high adherence to the Mediterranean dietary pattern had a lower risk of type 2 diabetes [73]. Yilmaz et al. (2015) examined the association between MC4R rs17782313 polymorphism of overeating and depression, and found that BMI was associated with the rs17782313 C allele. Moreover, the authors also noted that rs17782313 was significantly associated with depression and overeating behaviors, and that MC4R leads to weight gain and BMI via depression and overeating behaviors [42]. In the present study, a significant relationship was observed between individuals with CC genotype and consumption of an UDP with depression, while this association was not observed among individuals with CT genotype, despite an increase in OR. This may be because when a dangerous allele is placed next to a harmless allele, they behave differently than when two dangerous alleles are placed next to each other. Also, no significant relationship was observed between MC4R gene interaction and HDP and depression, but a logical inverse relationship was observed between CT genotype and HDP on depression. This may be because most people in the study chose an UDP as their dominant diet, and therefore, the interaction between the MC4R gene and an UDP is more closely linked to depression.

Several limitations must be considered when interpreting the results of the present study. Indeed, the cross-sectional design precludes causal inferences into the interaction between dietary intake and depression in individuals at risk for the MC4R gene allele. Another limitation is that the FFQ for evaluating the diet for accurate reporting is dependent on people’s memory and can be reported incorrectly. Further, based on the study population, this study is only generalizable to overweight and obese women, so it cannot be used for men and women with normal weight. Future research should take into account another limitation—depression-induced changes in eating behavior.

## Conclusion

Overall, these findings underline the importance of the UDP in the alarming prevalence of the depression in developing countries. Although the theoretical models of genetic-dietary patterns-depression interactions clearly need a more empiric foundation, the evidence from this study putatively shows a direct link between the incidence of depressive for people with CC alleles of MC4R gene and greater adherence to an unhealthy dietary pattern. The findings of this study show that the interaction of MC4R variants between individuals and high consumption of UDP can play an important role in the development of depression.

## Data Availability

The data that support the findings of this study are available from Khadijeh Mirzaei but restrictions apply to the availability of these data, which were used under license for the current study, and so are not publicly available. Data are, however, available from the authors upon reasonable request and with permission of Khadijeh Mirzaei.

## Abbreviations

ANCOVA: analysis of covariance
BMI: body mass index
BP: Blood pressure
CVD: cardiovascular disease
DBP: diastolic blood pressure
DASS: Depression Anxiety Stress Scales
FBS: Fasting blood sugar
FFM: fat free mass
FFQ: food frequency questionnaire
GLM: generalized linear model
HC: Hip Circumference
HDL-C: low density, lipoprotein cholesterol
IPAQ: international Physical Activity Questionnaire
LDL: low density lipoprotein
MC4R: melanocortin 4 receptor
PCA: principal component analysis
PCR-RFLP: polymerase chain restriction fragment length polymorphism
SBP: systolic blood pressure
SNP: single nucleotide polymorphism
TC: total cholesterol
TG: triacylglycerol
WC: waist circumferences.
